# Laparoscopic Management of Boerhaave’s syndrome: a case report with an intraoperative video

**DOI:** 10.1186/s12893-019-0576-7

**Published:** 2019-08-13

**Authors:** Hager Aref, Tahir Yunus, Obadah Alhallaq

**Affiliations:** Department of Surgery, International Medical Center, P.O. Box 2172, Jeddah, 21451 Saudi Arabia

**Keywords:** Boerhaave’s syndrome, Esophageal rupture, Spontaneous rupture, Laparoscopy

## Abstract

**Background:**

Boerhaave’s syndrome involves a sudden elevation in the intraluminal pressure of the esophagus, causing a transmural perforation. It is associated with high morbidity and mortality. Its treatment is challenging, and early surgical intervention is the most crucial prognostic element.

**Case presentation:**

We present a case of a 32 year-old male who presented after severe emesis with an acute onset of epigastric pain. He was diagnosed with Boerhaave’s syndrome. Displaying signs of shock mandated immediate surgical exploration with laparoscopic primary repair.

**Conclusion:**

The golden period of the first 24 hrs of the event still applies to cases of esophageal perforation. The scarcity of these cases makes a comparison between the various treatment methods difficult. Our data support the use of laparoscopic intervention with primary repair as the mainstay of treatment for the management of esophageal perforation.

**Electronic supplementary material:**

The online version of this article (10.1186/s12893-019-0576-7) contains supplementary material, which is available to authorized users.

## Background

Boerhaave’s syndrome has long been discussed in the medical literature. Dr. Herman Boerhaave described the first case in 1724 [1]. In 1946, Dr. Barrett performed the first successful operation for this rare condition [2].

Boerhaave syndrome or spontaneous rupture of the esophagus is a perforation of the esophagus that results from an abrupt increase in intraesophageal pressure along with negative intrathoracic pressure; for example, after forceful retching or severe vomiting. Esophageal perforation is a rare entity, with an incidence of 3.1 per 1,000,000 per year [3]. Among all esophageal perforations, about 15% are spontaneous perforations [4]. Boerhaave syndrome is associated with high morbidity and mortality. It can be fatal if left untreated. Its vague presentation may contribute to a delay in diagnosis and results in poor outcomes [5, 6]. Hence, early surgical intervention is the most important prognostic factor. The standard surgical treatment was through an open surgical approach, making the case even more challenging to manage in terms of postoperative recovery.

Herein, we present a case of Boerhaave’s syndrome, in which laparoscopic repair was successfully performed. In this paper, the authors support the use of laparoscopy for the primary repair of esophageal perforations as the mainstay of treatment. We present this case report to emphasize on the favorable outcome of using laparoscopy in the management of such cases, with an illustration of the intraoperative technique utilized in this case (Additional file 1: Video S1).


**Additional file 1:** A video showing Intraoperative findings and techniques, (Boerhaave’s Syndrome, primary repair of an esophageal perforation). (MP4 355452 kb)


## Case presentation

We report the case of a 32 year-old male patient who presented to our Emergency Department with acute onset of unbearable epigastric pain after severe emesis following food ingestion. The pain was radiating to his chest and back and increases by any movement. Due to severe pain and dyspnea, the patient was unable to lay supine insisted on remaining sitting up for the examination. He had an episode of hematemesis after the start of pain. His medical, family, and psychosocial history were irrelevant. He had no previous surgeries performed. Physical examination revealed an anxious patient who looked pale, and dehydrated. His vital signs were, a temperature of 37.5 C, heart rate of 103 beats/minute, and the rest were unremarkable. Examination of the cardiopulmonary system was unremarkable. Upon examining the abdomen, the epigastric area was tender with guarding in the upper abdomen but soft at the lower part. No rigidity was appreciated.

**Fig. 1:**
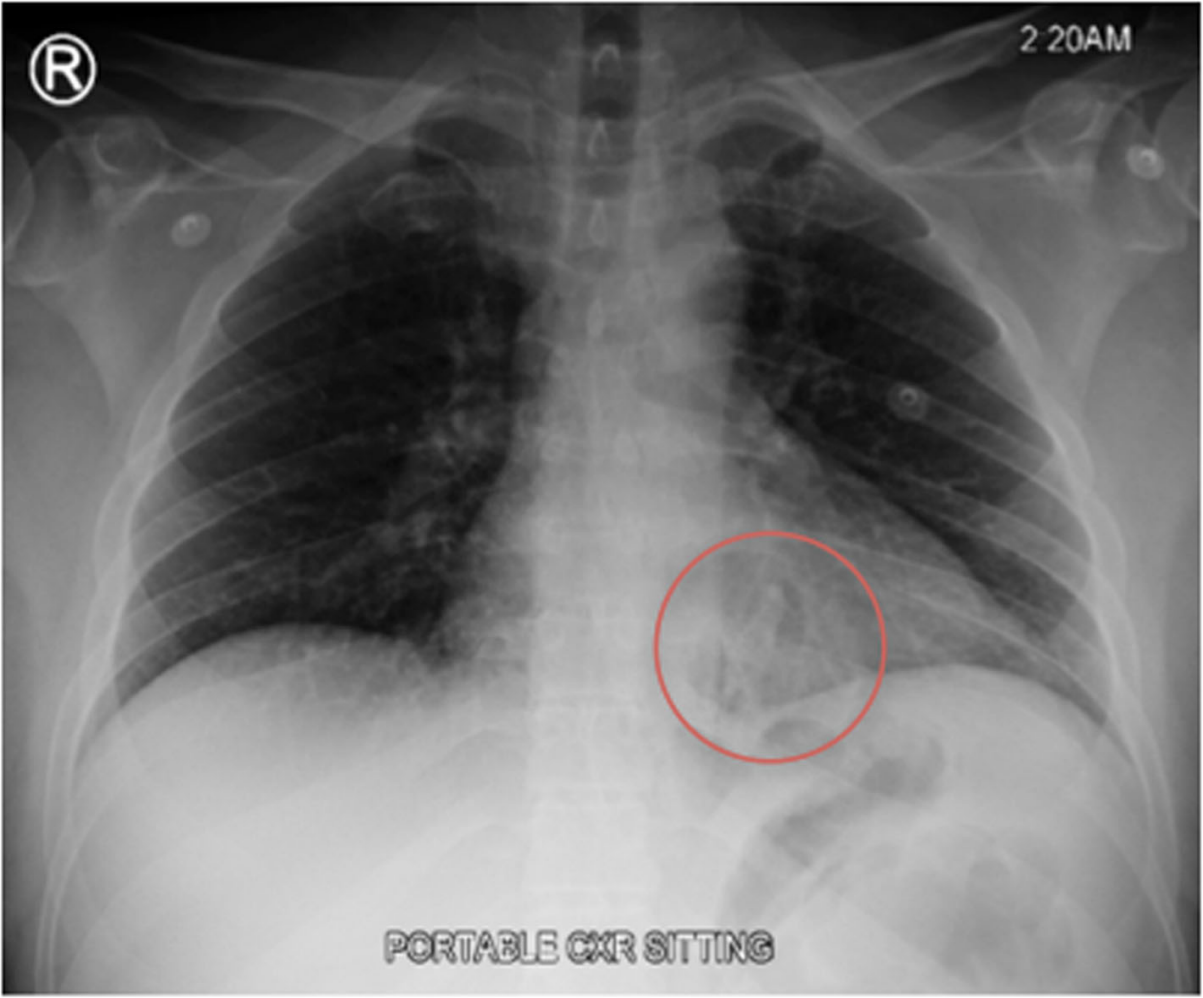
A Chest Radiograph showed a small air pocket in the paraspinal space

**Fig. 2:**
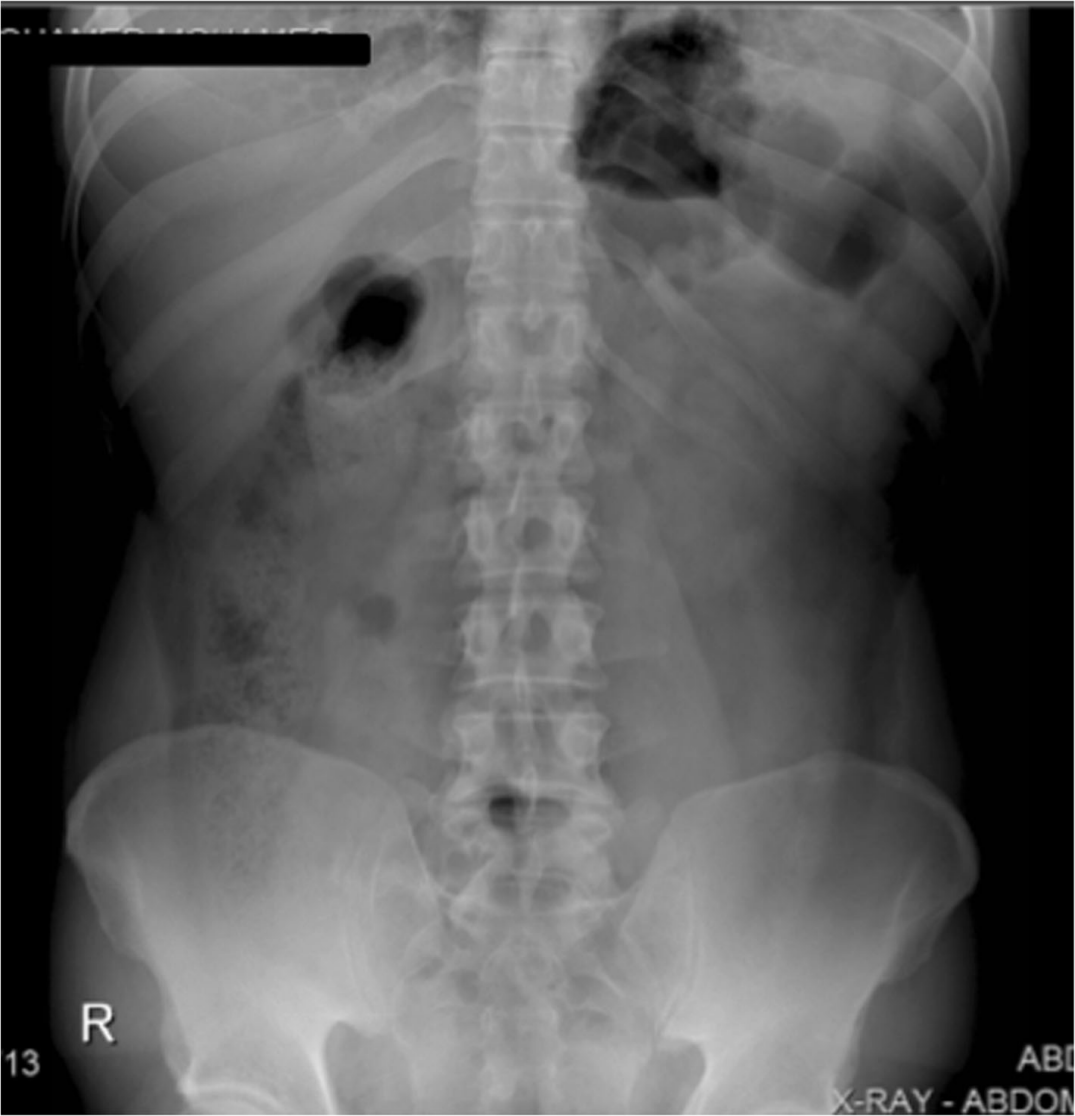
An abdominal Radiograph showed no intraperitoneal free air, otherwise an unremarkable x-ray

His laboratory investigations showed leukocytosis, WBC was 22 × 109/L. An electrocardiogram was done and showed sinus tachycardia. The chest (Fig. 1) and abdominal x-rays (Fig. 2) showed tiny air collection in the paraspinal space on the left side above the diaphragm, but no free air under the diaphragm and no evidence of pleural effusion.

**Fig. 3:**
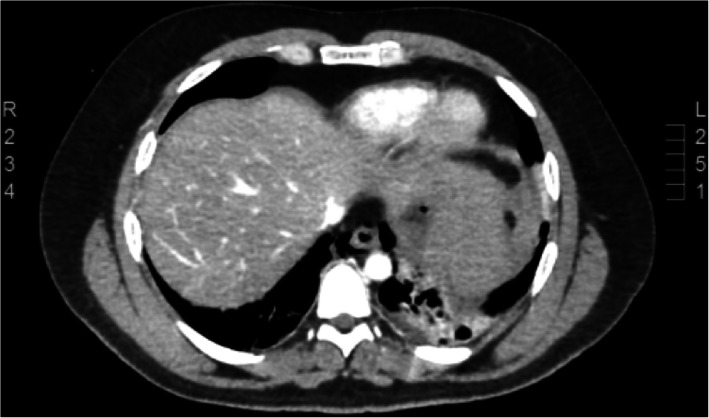
A CT Abdomen and pelvis, showed esophageal rupture at the gastro-esophageal junction with fundal intramural hematoma

He was admitted to the Intensive care Unit and underwent CT of the abdomen and pelvis, which revealed air around the distal esophagus with apparent thickening of the wall of the esophagus, the air was noted at the gastroesophageal junction with air bubbles in the gastro-hepatic ligament in the abdomen. A large intramural hematoma is noticed within the gastric fundus and basal infiltration of the left lung. Features were highly suggestive of rupture of the distal esophagus at the gastroesophageal junction with pneumo-mediastinum (Fig. 3).

The diagnosis of Boerhaave’s Syndrome was made. Intravenous antibiotic and fluid hydration were initiated. With signs of peritonitis, hematemesis, and impending shock, he was intubated and taken for laparoscopic abdominal exploration with the possibility of thoracic exploration. As a result, Thoracic surgery team was consulted and involved in the course of management of the case.

**Fig. 4:**
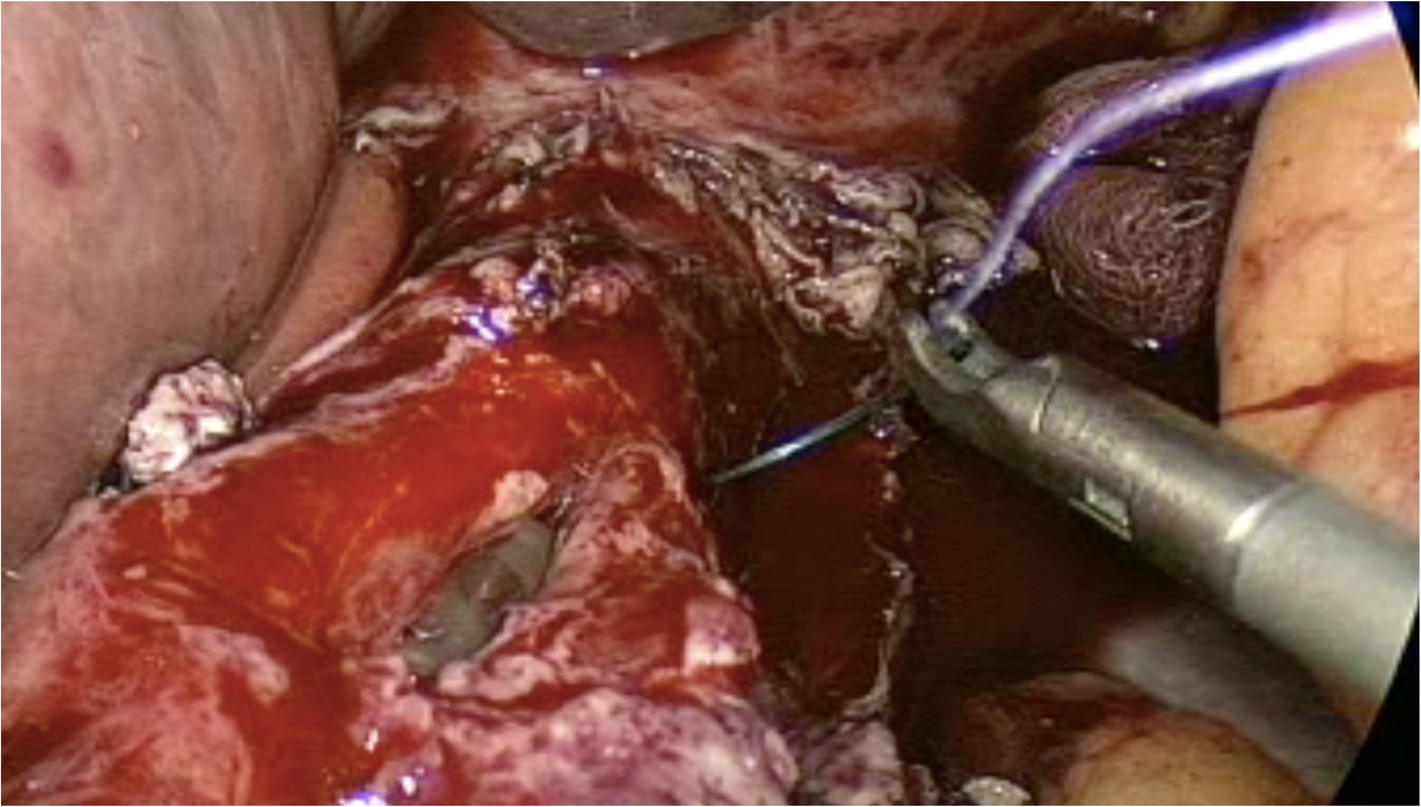
Intraoperative findings: a perforation in the lower third of the esophagus at the left posterior wall, measuring about 2 cm

**Fig. 5:**
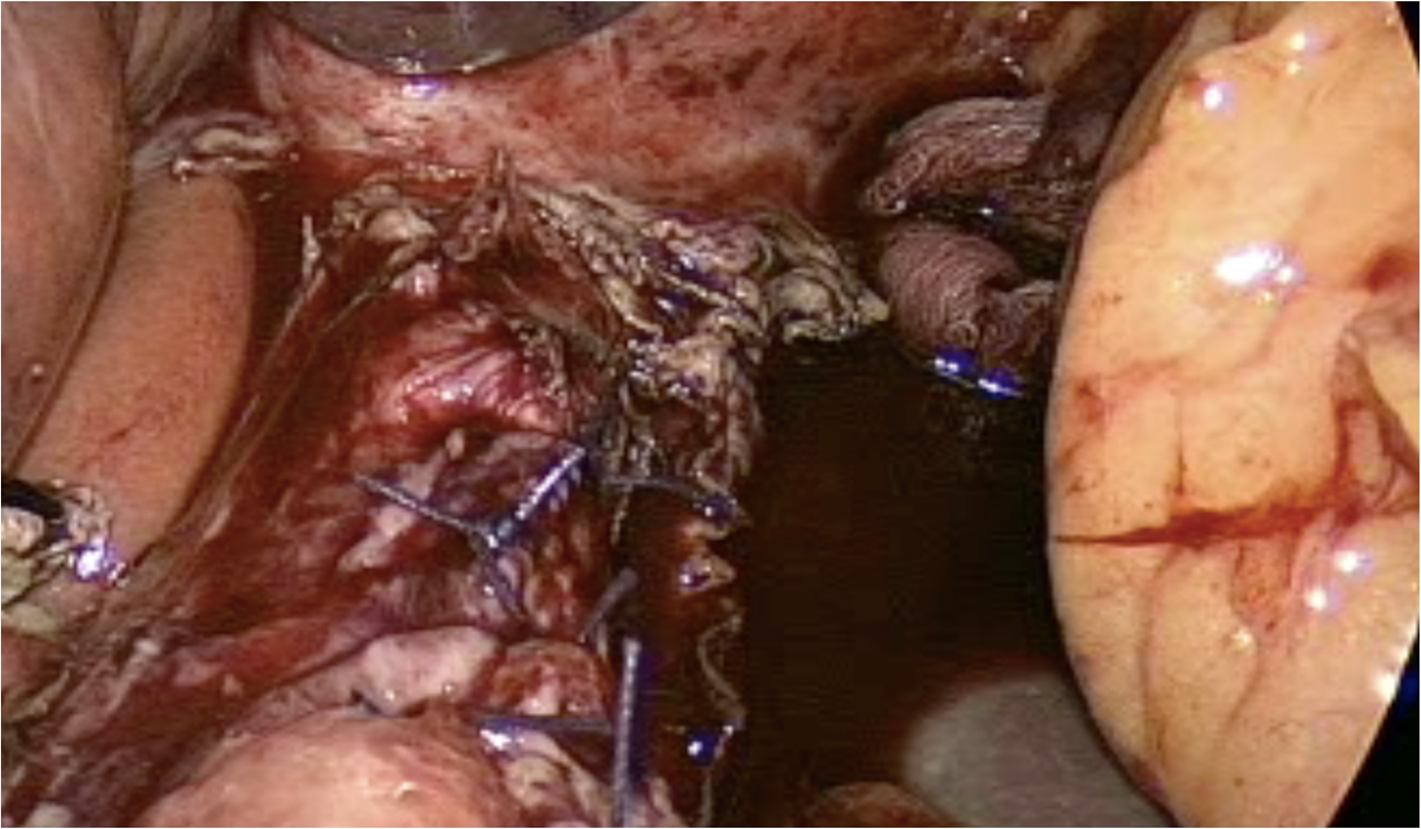
Intraoperative view following primary repair of the perforation

Upon entering the abdominal cavity, a generous hematoma was identified in the gastrosplenic ligament and filling the left upper quadrant area. No signs were indicating the presence of an abscess collection, nor a mass. Followed by dissecting through the pars flaccida on the right side of the stomach. The left side dissection initiated by cutting the greater omentum using a smart bipolar (Ligasure) device. The left crus of the diaphragm was identified, and the anatomy of the gastroesophageal.

(GE) junction was elaborated. We identified a 2 cm longitudinal perforation within the lower third of the esophagus at the posterolateral wall (Fig. 4). We continued the circumferential dissection of the esophagus, preserving the Posterior Vagus nerve. A 38-French gastric calibration tube was carefully advanced across the GE Junction into the stomach. The edges of the esophageal perforation were cleaned, and primary repair was performed with interrupted 2–0 polyglactin (Vicryl) stitches, (Fig. 5). Then, an omental patch was sutured over the perforation area (Additional file 1: Video S1).

A feeding jejunostomy was inserted to gain enteral access for postoperative nutritional support, and a nasogastric tube was advanced into the stomach under intraoperative guidance. Drains were placed intrabdominal and posterior mediastinal.

The patient was then shifted to the Intensive Care Unit. He was kept on the ventilator post-op and extubated the following day. He was kept on antibiotics and was started on jejunostomy feeding.

**Fig. 6:**
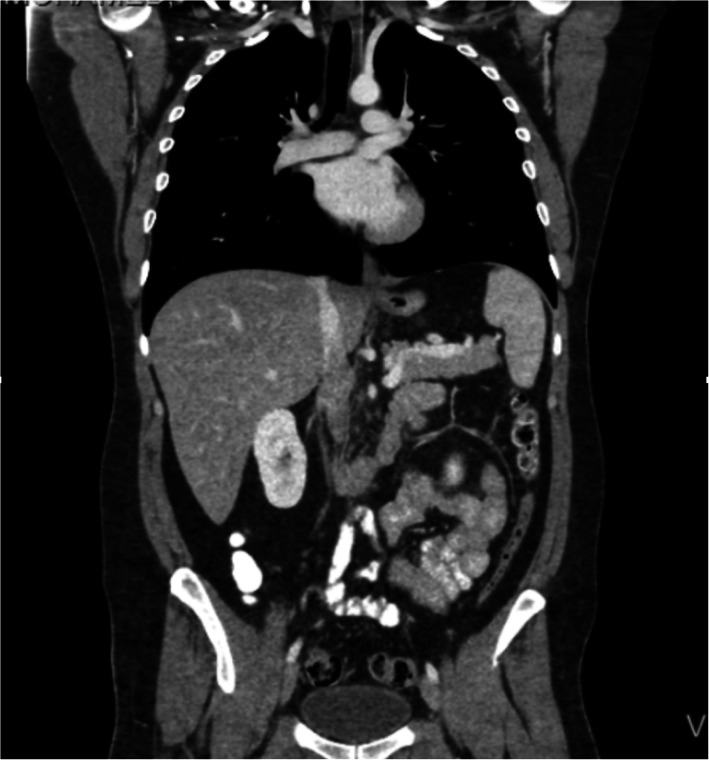
CT of the chest, abdomen, and pelvis performed in the postoperative setting, showing no evidence of a leak or significant collection

On postoperative day three, he was transferred to the regular surgical floor. Over a week duration, his leukocytosis resolved. On postoperative day 4, the patient was started on a clear fluid diet, which was well tolerated. One week postoperative, a CT scan of the abdomen-pelvis (Fig. 6) was performed and confirmed the absence of fluid collections and contrast leak. He was discharged home on postoperative day eight but represented in the emergency room after 2 days with upper back and chest pain that aggravated with respiration. Examination of the chest revealed, left lower chest crepitations. He was afebrile and had a normal leukocyte count. A chest x-ray was done showing pleural effusion, for which he was re-admitted for further management. Upon admission, a repeat CT scan of the abdomen did not show any signs of contrast leakage and confirmed a left basal pulmonary consolidation and minimal left-sided pleural effusion. Pleural tapping with fluid analysis was performed, and he was managed with antibiotics and chest physiotherapy, which facilitated the resolution of the pleural effusion. He was discharged home in good condition after a week of hospitalization.

He was reassessed in the surgical clinics and was doing very well with no complains. An upper endoscopy was performed 2 months after the surgery and confirmed normal esophageal and stomach anatomy.

## Discussion

Boerhaave’s syndrome refers to spontaneous esophageal transmural rupture [7]. Boerhaave’s Syndrome has a mortality rate of 20–40%, which is considered the highest among all etiologies of esophageal perforation [8, 9]. That goes back to the fact that perforation leads to the leak of acids, enzymatic juices, and micro-organisms, which is causing widespread mediastinal & pleural contamination [10].

To diagnose this condition, physicians need to have a high index of suspicion. Although, there has been a publication where the classical findings of Boerhaave’s syndrome, was described as aiming to help in making the diagnosis. These findings were named as Mackler’s triad, and it includes emesis or retching, chest pain, and subcutaneous emphysema. The author has mentioned in his article that, these signs might not all be present in one case [11]. In the case we are presenting, he presented with severe chest pain, difficulty breathing and emesis, but subcutaneous emphysema was not identified. This condition can be confused with other entities such as perforated peptic ulcer, myocardial infarction, and acute pancreatitis, leading to the delay in diagnosis [12]. Hence the high mortality chance of 39% [13]. All the aforementioned entities were excluded in the presented case by clinical examination, laboratory and imaging investigations.

While having the suspicion of Boerhaave’s, radiological studies should be performed to aid confirm the diagnosis and plan surgical intervention. These include plain chest x-ray, which is found to be abnormal in more than 90% of cases, with free air present in the mediastinal or peritoneal cavity [14]. Less often, with some cases where cervical esophageal perforations are suspected, a person may find prevertebral or subcutaneous air on x-ray. Interestingly, those signs were also evident in the described case. Despite the high prevalence of abnormal findings on a chest x-ray, CT scan of the chest and upper abdomen with oral contrast has a better diagnostic advantage and is considered as the preferred diagnostic imaging modality.

The appropriate method for the management of esophageal perforation depends on many factors, such as the severity, time since perforation, the location, age, and status of the patient upon presentation. In such serious condition, surgery is considered as the mainstay treatment, and the outcome depends on early diagnosis, and treatment. The duration between the onset of perforation and surgery should not influence the decision of performing a primary repair.

The literature reported that primary repair of the esophagus complemented by mediastinal and chest drainage is associated with a 90% success rate. This is especially true in cases where the esophageal rupture is diagnosed early (within 24 h) with is no associated esophageal diseases [15–17].

If the diagnosis was late (more than 24 h), the outcome was found to be significantly worse, in which operative management is less successful [18, 19].

Recent studies have recommended primary repair for esophageal regardless of the time interval between perforations [20, 21] and surgery [22].

There have been few reports describing the laparoscopic management of this entity. Studies showed that laparoscopic surgery has many advantages over open surgery, which are a shorter length of stay, faster bowel recovery and earlier mobilization, and less blood loss [23, 24].

The trans-hiatal approach results in a shorter length of hospital stay. In a study by Danson et al., the mean length of stay in the laparoscopic approach was 14 days [13]. In contrast, patients who underwent opposed open thoracotomy and washout with primary repair had a longer mean of 20.5 days. In patients who presented the following 48 h and underwent primary closure over a T-tube, the mean length of stay was longer than the previous group, which is 35.7 days [6]. There were no mortalities in cases managed by laparoscopic trans-hiatal repair, however open thoracotomy and primary repair was associated with a high mortality rate of 20%. These results, strongly support laparoscopic management of this condition. Herein we present an example of successful laparoscopic treatment of a case of Boerhaave’s syndrome, which involved primary surgical repair of the perforation. Despite the fact that it was a late presentation; exceeding 48 h from onset of pain, it had a considerably better outcome as compared to other cases reported in the literature. Furthermore, the total length of stay was significantly less than what was previously reported in the literature of similar cases (total LOS was 8 days for this case, in comparison to 14 days in the literature). This outcome signifies the success of laparoscopic primary repair of esophageal perforation.

## Conclusion

The golden period of the first 24 h of insult still applies for cases of esophageal perforation. The rarity of these cases makes a comparison between the various methods of treatment difficult. The data presented support the use of laparoscopic operative intervention with primary repair as the mainstay of management of similar esophageal pathology, which showed success even with late presentations. The authors further emphasize the importance of involving a multidisciplinary team in the management of such challenging cases. This includes Intensive care physician, Thoracic surgeon, and laparoscopic surgeon.

## Data Availability

All the data presented is contained within the manuscript file.

## Abbreviations

CT: Computed Tomography
ER: Emergency Room
GIT: Gastrointestinal Tract
HIS: Health Information System
ICU: Intensive Care Unit
MRI: Magnetic Resonance Imaging
